# Crystal ball – 2013

**DOI:** 10.1111/1751-7915.12014

**Published:** 2012-12-18

In this feature, leading researchers in the field of environmental microbiology speculate on the technical and conceptual developments that will drive innovative research and open new vistas over the next few years.
