# Supplementary material for: Crystal ball – 2013
Source: Microb Biotechnol. 2012 Dec 18;6(1):3–16. doi: 10.1111/1751-7915.12014 (PMC3815379; doi:10.1111/1751-7915.12014)
Supplement: Related texts

# Article commentary: The original syn

**Published:** 2012-12-18

**Authors:** Tom Curtis

**Affiliations:** School of Civil Engineering and Geosciences, Newcastle UniversityNewcastle upon Tyne, NE17RU, UK

No review of the power of synthetic biology is complete without a sage affirmation that the fight for environmental protection will be immeasurably served by synthetic microorganisms. Yet, to state the obvious, environmental protection occurs in the environment and synthetic biology occurs in the test tube. Culture and culturability are the original sin of synthetic biology. The field of microbial ecology has blossomed under the insight that the Petri dish is but a pale and partial shadow of real microbial environments. Yet synthetic biologists, who seek to do better than evolution, use that tiny fraction (lets say 0.0001%) of evolutions handiwork that will grow in the lab.

The implicit assumption is that in many cases it will be easier, or indeed absolutely necessary, to meet an environmental need, by creating novel a life form in the lab rather than using some pre-existing organism ‘off the peg’. This is an assumption that is far more likely to be met if you only consider the organisms that grow in the lab and ignore competing philosophies such as microbial resource management.

However, this ‘diversity blindness’ is not the biggest issue. Indeed it would require churlish counter-hubris to assume that someone somewhere could not engineer an organism with a useful function not found in nature. The question is how to deploy it. There is little evidence that we can get an organism from the laboratory into the field in any kind of systematic way. Those mindful of the fears of rampant microbes have spoken of forms of genetic containment, sequencing tweaks that would ensure the demise of a released but unwanted organism. They hardly need bother. Genetically manipulated (and I suspect cultured organism in general) seem to simply die away when faced with the rigours of the environment. Containment is problematic. Mark van Loosdrecht has pointed out that a sterile and sterilizable reactor (i.e. a very big test tube) will cost 200 times more than an equivalent open reactor. Moreover, the feedstock for such a reactor, presumably the waste itself, would need to be sterilized before use. Immobilization and protection behind membranes will be no less expensive.

The simple truth is that it that virtually no one has successfully introduced a cultured or genetically engineered organism into an environment. Moreover, we have absolutely no idea why this is problematic. Those with the courage to admit this might be a problem speak of the need for more robust organisms. However, there is no evidence that the manipulated organisms some how have flimsy phospholipids or inherently inadequate cell walls. Indeed the focus on the individual organism betrays the mindset of the test tube where a single organism can be considered in isolation. In the real world (and we have known this since RA Fisher coined his fundamental theorem) fitness is a function of both the properties of the organism and the properties of all the other organisms it might encounter. This means that the design of an organism to accomplish a function in an open environment must consider not only the function, but also executing that function in the context of the possibly thousands of other species it might encounter. This is, to put it mildly, a tricky problem and there is, at present, no theoretical framework for doing this. If it is resolvable, and it may be, resolution will come when synthetic biology gets out of the test tube and embraces ecology and evolution. This may require advances in all three domains.

Which brings me to my final point. If these problems are well known and the solutions are not. Why do so many people continue to assert that they will solve a problem which they no idea how, or perhaps willingness, to address? In private, this question will be greeted with a cynical shrug and the implication that this form of debasement is the price you pay for funding. But this is dangerous, for what might start as naïve optimism and then fade into grant-getting gamesmanship can transmogrify into a moral and intellectual fraud. Defrauding us of not simply money, but that far more precious resource: time. We really do need to radically change environmental biotechnology and we do not have forever to do it. So if you want to do this using synthetic organisms: get out of the test tube or get out of the way.

# Article commentary: Recombination-based DNA assembly in metabolic engineering: a goodbye to old workhorses?

**Published:** 2012-12-18

**Authors:** Jean-Marc Daran, Jack T Pronk

**Affiliations:** Department of Biotechnology, Delft University of Technology and Kluyver Centre for Genomics of Industrial FermentationJulianalaan 67, 2628, BC Delft, The Netherlands

For the better part of four decades, genetic engineering has relied on a universal toolbox containing three indispensable implements: restriction enzymes, ligases and, last but not least, *Escherichia coli*. Even those of us who now languish behind laptops and in meetings rather than work at the bench instantaneously recognize the smells of Luria broth and plasmid preps.

Since the hallmark paper of Cohen and colleagues (1973), many elegant variations on the theme of restriction and ligation have been introduced to make *E. coli*-based cloning tremendously versatile. However, especially in the field of metabolic engineering, two major limitations of this trusted approach gradually became apparent. First, as the complexity of the desired DNA constructs increased, design of multi-gene constructs based on restriction and ligation turned into a molecular biologist's equivalent of Rubik's cube and the construction itself into a multi-step, painstaking and time-consuming exercise. Even 3 years ago, most of us would not have contemplated the assembly of, for example, a 20-fragment expression vector, neither as part of a PhD or postdoc project, nor in an industrial research setting. Second, the multi-step nature of DNA assembly via restriction and ligation and its requirement for unique restriction sites complicated the implementation of combinatorial approaches in the assembly of complex constructs, for example to optimize expression of a heterologous pathway by testing various combinations of enzymes from different donor organisms or by testing different combinations of promoter fragments in a multi-enzyme, heterologously expressed pathway.

During the past 4 years, fast developments in synthetic biology led to the (re)discovery and optimization of a powerful alternative approach for assembling DNA fragments that is entirely independent of restriction and ligation. This approach is based on homologous recombination of short, shared terminal sequences of the linear DNA fragments that need to be assembled and can be subdivided into *in vitro* and the *in vivo* methods.

*In vitro* methods for DNA assembly by recombination depend on cell-free systems, in which recombinase enzyme(s) take care of the assembly (see e.g. Gibson *et al*., 2010a). The major advantage of this method over restriction and ligation is that it enables one-step assembly of multi-fragment constructs, completely independent of the availability of unique restriction sites. The required short stretches of sequence overlap can easily be introduced, either by PCR amplification of target sequences with ‘overhanging’ primers or by the increasingly cost-efficient process of DNA synthesis. After *in vitro* assembly of a construct, it is transformed to a suitable microbial host, in many cases still *E. coli*.

The *in vivo* methods are based on the same principles of generation and recombination of homologous termini as the *in vitro* methods. However, they go one important step further by eliminating the need for separate assembly and transformation steps. The *in vivo* methods rely on the high efficiency of the cellular homologous recombination machinery of some microorganisms by simply transforming the required cocktail of linear DNA fragments into the host cell. When a suitable origin of replication and selection marker are included among the fragments, the transformed microorganism then faithfully recombines the fragments into an autonomously replicating episome, without any prior enzymatic treatment. *In vivo* recombination-based DNA-assembly platforms have been developed in *Bacillus subtilis* (Itaya *et al*., 2008) and in specific *E. coli rec* mutants (Datsenko and Wanner, 2000; Li and Elledge, 2005). However, these bacterial systems have a limited capacity to efficiently assemble multiple fragments and are outperformed by the incredible recombination performance of the star player of *in vivo* assembly, the yeast *Saccharomyces cerevisiae*.

*In vivo* assembly of DNA fragments in yeast was already proposed and demonstrated 30 years ago (Orr-Weaver *et al*., 1981; Kunes *et al*., 1987; Ma *et al*., 1987). However, it failed to really take off as a mainstay DNA construction method, perhaps mainly due to the absence of efficient techniques to generate the required homologous sequences. For a long time, its application was largely limited to the cloning of large DNA fragments that were difficult to manipulate by restriction and ligation (Larionov *et al*., 1996). *In vivo* assembly in *S. cerevisiae*, also known as transformation-associated recombination (TAR), only really caught the spotlight and took off when it was elegantly applied in the complete chemical synthesis, assembly, and cloning of a synthetic *Mycoplasma* genome by Gibson and colleagues (2010b). This milestone achievement was accompanied by a thorough evaluation of the high-fidelity assembly properties of *S. cerevisiae*, which convinced many metabolic engineers, especially those already in the yeast field, to use this new molecular biology platform for the construction of large and complex metabolic pathways (Merryman and Gibson, 2012).

As yeast biologists, we have been amazed by the power and simplicity of recombination-based assembly in our ‘pet’ organism when we introduced the technique into our lab. In addition to accelerating construction of unique, multi-gene constructs, the technology facilitates combinatorial approaches in metabolic engineering and, due to its one-step simplicity, is highly compatible with automated, high-throughput strain construction. Gazing in the crystal ball, we would never dare to predict the complete demise of good old *E. coli* as a molecular biology workhorse. However, we hope and predict that, in the coming years, this simple technique for DNA assembly will find its way into many labs and especially into those that do not have a tradition in yeast molecular genetics.

## References

[b1] Cohen SN, Chang ACY, Boyer HW, Helling RB (1973). Construction of biologically functional bacterial plasmids *in vitro*. Proc Natl Acad Sci USA.

[b2] Datsenko KA, Wanner BL (2000). One-step inactivation of chromosomal genes in *Escherichia coli* K-12 using PCR products. Proc Natl Acad Sci USA.

[b3] Gibson DG, Smith HO, Hutchison CA, Venter JC, Merryman C (2010a). Chemical synthesis of the mouse mitochondrial genome. Nat Methods.

[b4] Gibson DG, Glass JI, Lartigue C, Noskov VN, Chuang RY, Algire MA (2010b). Creation of a bacterial cell controlled by a chemically synthesized genome. Science.

[b5] Itaya M, Fujita K, Kuroki A, Tsuge K (2008). Bottom-up genome assembly using the *Bacillus subtilis* genome vector. Nat Methods.

[b6] Kunes S, Ma H, Overbye K, Fox MS, Botstein D (1987). Fine structure recombinational analysis of cloned genes using yeast transformation. Genetics.

[b7] Larionov V, Kouprina N, Graves J, Chen XN, Korenberg JR, Resnick MA (1996). Specific cloning of human DNA as yeast artificial chromosomes by transformation-associated recombination. Proc Natl Acad Sci USA.

[b8] Li MZ, Elledge SJ (2005). MAGIC, an *in vivo* genetic method for the rapid construction of recombinant DNA molecules. Nat Genet.

[b9] Ma H, Kunes S, Schatz PJ, Botstein D (1987). Plasmid construction by homologous recombination in yeast. Gene.

[b10] Merryman C, Gibson DG (2012). Methods and applications for assembling large DNA constructs. Metab Eng.

[b11] Orr-Weaver TL, Szostak JW, Rothstein RJ (1981). Yeast transformation: a model system for the study of recombination. Proc Natl Acad Sci USA.

# Article commentary: The future of sustainable fish production lies in vaccine research and development and revised regulatory measures

**Published:** 2012-12-18

**Authors:** Joachim Frey

**Affiliations:** Institute of Veterinary Bacteriology, Universität BernLaenggass-Str. 122, Postfach, CH, 3001, Bern, Switzerland

A significant amount of proteins and high-quality lipids in human nutrition originates from fish and shellfish. As a result of the increasing human population and its improving health and welfare, most wild fish species have become overexploited by overfishing. The world's fish populations therefore suffer from dramatic declines. Breeding fish in analogy to breeding domestic ruminants, pigs and poultry will significantly protect endangered wild fish populations and can eventually re-establish an equilibrium of aquatic life. However, this can only be achieved by cultivating more non-carnivorous species and by changing the food requirements of carnivorous species by supplementing their nutrition with correctly processed slaughter waste materials and vegetable products. Furthermore, fish farming must not adversely affect the aquatic and terrestrial equilibrium by emissions of waste products and other pollutants.

Worldwide aquaculture has increased at an average of 9.5% per year over the past 30 years, compared with 2.6% for terrestrial meat production, freshwater production representing the highest increase. Salmon production in Norway by aquaculture, for example, has, in less than three decades, increased from virtually zero to over one million tons in 2011. In 2011 the world fisheries production of freshwater fish amounted to 11.5 megatons captured versus 44.3 megatons cultured; of marine fish 78.9 megatons captured versus 19.3 megatons cultured; and of crustaceans 6.1 megatons captured versus 5.3 megatons cultured (FAO, 2012). This leaves a vast potential for future development in aquaculture.

As with intensive animal breeding, aquaculture is strongly hampered by infectious diseases which cause massive economic losses and ecological damage. In shrimp production, a mere 10% of the juvenile shrimps leaving the hatchery reach the age of breeding maturity whereas 90% succumb mainly to infections. This leads to mostly uncontrolled use of huge amounts of antibiotics which represent a high risk to human and animal health and cause enormous environmental damage. In fish culture, antibiotics are primarily administered as medicated food whereby 70–80% of the active substances end up in the environment via food surplus and residual substances excreted from fish. Although there are no clear data on the worldwide use of antibiotics in aquaculture, estimations vary from 300 g to 1.5 kg per ton of fish or shellfish produced. This causes a significant pollution of surface waters leading to ecotoxic effects, impact on natural microbial communities and a large-scale emergence of antibiotic resistant and multi-resistant human and animal bacterial pathogens, in particular of the groups of *Vibrio*, *Aeromonas* and various *Enterobacteriaceae*. Antibiotic resistant pathogens are expected to be the main cause of unsuccessful disease therapy in human medicine in the near future, and this will probably throw back the current successful high public health status in most countries for decades. Hence a major goal that must be reached in agriculture and in aquaculture, in particular, is a drastic cutback or preferentially a ban of the use of antibiotics.

The development of adequate vaccines and vaccine application procedures for aquaculture will represent a major challenge in the next two decades in order to achieve the goal of a sustainable fish production. The feasibility of controlling furunculosis, the major infectious disease in farmed salmon, caused by the bacterium *Aeromonas salmonicida* subsp. *salmonicida*, by means of vaccines, has been demonstrated in Norvegian salmon production. In 1988, with an annual production of 50 000 tons of salmon, 50 000 kg of antibiotics were used per annum. In 2008 with a production of 850 000 tons of salmon – a 17-fold increase – the use of antibiotics was reduced by a factor of 50 to around 1000 kg per year after the successive introduction of four generations of vaccines between 1989 and 1999 (Stevens, 2011). This example shows the efficacy of vaccines in attempts to reduce or ban altogether the use of antibiotics in aquaculture.

While vaccines against furunculosis of salmon present a most successful example of preventive medicine of farmed fish, vaccines and vaccine application methods for most other fish or against most other diseases are still lacking or have low efficacy. The complex nature of the aquatic microbial world, the relatively poor knowledge of the fish immune system and of molecular mechanisms of pathogen–host interaction of many fish diseases require considerable efforts in basic research in order to yield new concepts for fish vaccines. Whereas basic research mostly relies on financing by the public sector, investments by the private sector for the development of new vaccines, novel vaccine formulae, more efficient and less nocent adjuvants as well as for novel rational vaccination procedures such as oral vaccination or immersion vaccination are essential to attain the aim of improved fish health and consequently a more sustainable aquaculture. In order to attract such important investments in the relatively small economic sector of animal health, regulatory procedures that govern registration and licensing of animal vaccines must be simplified and revised to make them affordable for a market with low profit margins. Currently, maximal production costs for vaccines for farm animals and large fish (where individual vaccination is applied) is around €0.10 per dose, whereas that for small fish is estimated to be 100 times lower. In comparison, costs for the registration of a new animal vaccine in Europe currently amount to around one to two million Euro, not including the costs for the preparation of the registration documentation. High costs of administration and regulatory measures often discourage the private sector from investing in the development of novel and more efficient animal vaccines, leaving old, often rather inefficient vaccines on the market or offering no solutions to combating emerging animal epidemics, even though, in many cases, public sector research has provided the necessary basic molecular knowledge.

In order to attain the goals needed for sustainable fish production in the future, significant inputs into exciting projects on fish immunology and pathogen–host interaction of fish disease are expected from microbiologists, valuable investments are expected from the private sector and unbureaucratic and efficient procedures are expected from the regulatory authorities.

## References

[b201] FAO (2012).

[b202] Stevens C (2011).

# Article commentary: Gleaning and assembling omics parts lists from soil

**Published:** 2012-12-18

**Authors:** Janet K Jansson

**Affiliations:** Department of Ecology, Earth Sciences Division, Lawrence Berkeley National, Laboratory1 Cyclotron Road, Berkeley, CA, 94720, USA

Microbial ecologists are currently being inundated with increasing amounts of omics data, including millions of phylogenetic sequence reads (i.e. pyrotag or I-tag sequences), billions of base pairs of total nucleic acid sequences (i.e. metagenomes and metatranscriptomes), as well as thousands of peptides and metabolites from several types of environmental samples. Although this is a fantastic resource, the current challenge has been to glean relevant information from the data. Soil omics has proven to be particularly challenging due to the high microbial diversity inherent to most soils, combined with the difficulty in extraction of all macromolecules from the soil matrix.

That being said, I predict that within the next decade the bioinformatics and computing resources will have caught up to handle mega- and multi-omics datasets from soil. For metagenomics and metatranscriptomics datasets, the three major current limitations are the assembly, annotation and data mining steps. No soil metagenome has been sequenced with sufficient depth to enable assembly of more than a handful of draft genomes. One example is the recent successful assembly of a draft genome of a novel methanogen from permafrost soil (Mackelprang *et al*., 2011), but the microbial diversity in permafrost is lower than that in most other types of soil. Another example was the recent assembly of several draft genomes of novel species, including from phyla with no cultivated representatives, from uranium-contaminated sediments (Wrighton *et al*., 2012). Although a typical soil still has a higher diversity than permafrost or contaminated sediment, with sufficient sequencing depth now approaching terabases of sequence per sample, even soil should be possible to better assemble. It should also be possible to use binning approaches such as emergent self-organizing map analysis (ESOM) (Dick *et al*., 2009) that take into account read signatures, together with read coverage to bin reads into draft genome bins, as demonstrated for other sample types (Dick *et al*., 2009; Wrighton *et al*., 2012). In addition, I predict that we will have better functional gene databases that have been validated for screening the assembled data and raw reads. Also, it will be advantageous to have more omics data collected across time series and across environmental gradients, such as targeted in the Earth Microbiome Project (http://www.earthmicrobome.org).

Once we have access to well annotated soil metagenomes, the expression can be further validated by determining which genes are expressed and represented in metatranscriptomes, or translated into proteins and represented in metaproteomes. Having access to multi-omics data from the same samples should greatly increase our ability to gain a better understanding of the key roles that microbes play in different soil habitats and how those functional roles may be perturbed, for example by climate change. Already we are seeing examples of where different omics datasets have been combined and correlated for other types of environments, including marine (Mason *et al*., 2012) and the human gut (Erickson *et al*., 2012).

Finally, once we have this information more easily accessible and at our fingertips it will be possible to get answers to questions that have been eluding soil microbial ecologists to date including the following: (i) What microbial species and biochemical pathways are most important for cycling of soil nutrients, including carbon and nitrogen under given conditions? (ii) What microbial species and functions are perturbed by climate change, pollutants or other anthropogenic factors? (iii) What are the optimum combinations of bacteria, fungi, archaea, protists and viruses for a healthy soil that supports the growth of plants? and (iv) How are soil microbial community members and functional processes predictive of the potential for greenhouse gas emissions, bioremediation of pollutants and optimal crop growth? Eventually, this information should be relatively easy to obtain as sequencing costs continue to decline, computing facilities continue to increase, and databases continue to be updated.

## References

[b301] Dick GJ, Andersson AF, Baker BJ, Simmons SL, Thomas BC, Yelton AP, Banfield JF (2009). Community-wide analysis of microbial genome sequence signatures. Genome Biol.

[b302] Erickson AR, Cantarel BL, Lamendella R, Darzi Y, Mongodin EF, Pan C (2012). Integrated metagenomics/metaproteomics reveals human host-microbiota signatures of Crohn's disease. PLoS ONE.

[b303] Mackelprang R, Waldrop MP, DeAngelis KM, Chavarria KL, Blazewicz SJ, Rubin EM, Jansson JK (2011). Deep metagenome sequencing illuminates permafrost response to thaw. Nature.

[b304] Mason OU, Hazen TC, Borglin S, Chain PSG, Dubinsky EA, Fortney JL (2012). Metagenome, metatranscriptome and single cell sequencing reveal microbial response to Deepwater Horizon oil spill. ISME J.

[b305] Wrighton KC, Thomas BC, Sharon I, Miller CS, Castelle CJ, VerBerkmoes NC (2012). Fermentation, hydrogen and sulfur metabolism in multiple uncultivated bacterial phyla. Science.

# Article commentary: Merging multi-omics and microbial communities for robust biofuels production

**Published:** 2012-12-18

**Authors:** Adam Robbins-Pianka, Rob Knight

**Affiliations:** Department of Computer Science, University of Colorado at BoulderBoulder, CO, 80309, USA; Department of Computer Science, BioFrontiers Institute, Department of Chemistry and; Biochemistry, University of Colorado at Boulder, and Howard Hughes Medical InstituteBoulder, CO 80309, USA

Recent advances in metagenomics, metatranscriptomics and metabolomics techniques have enabled researchers to ask previously intractable questions about the biological world. Looking toward the future, it is clear that ‘multi-omics’ analyses that combine these three approaches, perhaps also including metaproteomics and single-cell or microculture genomics, will lead to important insights throughout microbial ecology. Rapid improvement in high-throughput sequencing technologies facilitates the analysis of larger and more complex microbial assemblages, and there is now great potential for advancements that impact our everyday lives.

In this respect, production of fuels from renewable sources will be increasingly important to our security, economy and environment. Microbes will play a central role in the robust synthesis of fuel from plants and algae. In particular, algae are an attractive candidate for mass-scale fuel production because they can grow rapidly on non-arable land. Already there are several companies capitalizing on this promising approach (e.g. Solix Biofuels, Sapphire Energy, Algenol Biofuels), and a more complete understanding of algal genomes, transcriptomes, and metabolomes will lead to higher yields and lower cost. However, monocultures are inherently susceptible to invasion, and this principle also applies to monocultures of algae. Building better models of individual species, and of the interactions of more stable multi-species assemblages, through an understanding of their interactions across expression levels is thus crucial.

Manipulation of biofuel strains on the genomic level, including mutants, knockouts and transgenics, is an important component of optimization of fuel production, but it is at present difficult to design a selection for improved biofuel producers, and screening techniques are still laborious. For individual strain improvement, transcriptional data, especially collected over time to assess response to perturbation and to multiple growth conditions, complemented by metabolomics analyses, would provide crucial data for improved systems biology models that seek to predict which alterations are most likely to increase overall efficiency of sunlight conversion, or enhance production of target molecules, e.g. advanced liquid biofuels such as butanol. For example, light to biomass conversion can be improved by making structural changes to reduce the antenna size in order to minimize overshading in liquid culture (Ort and Melis, 2011), but can also be improved by altering the regulation of light harvesting proteins (Beckmann *et al*., 2009). A better understanding of the regulatory and metabolic networks of target biofuel-producing algae could potentially provide numerous additional targets of the second type, especially when coupled with increasingly advanced metabolic reconstruction techniques that can incorporate multi-omic data (Yizhak *et al*., 2010).

However, an understanding of individual species is unlikely to be sufficient: designing communities that are robust to invasion has the potential to assist with problems scaling biofuels systems from laboratory to production scale. Diseases of algae are common, with pathogens including viruses, bacteria, fungi and other eukaryotes (Gachon *et al*., 2010). Individual strains of algae have strategies for inhibiting bacterial growth, such as by secreting compounds that interfere with bacterial quorum sensing (Rajamani *et al*., 2008), and the possibilities for manipulation of this type of inter-kingdom signalling are just beginning to be explored. Some phytoplankton also inhibit the growth of competing species under certain conditions (Prince *et al*., 2008), or of themselves, (Vardi *et al*., 2007), and these effects could also be manipulated (e.g. by moving normally autoinhibitory pathways into a different species to create allelopathic interactions). However, our ability to understand these effects in order to design robust synthetic communities are still very much emerging: even in bacteria, predicting mutualistic and antagonistic interactions is still challenging (Freilich *et al*., 2011), and algal genomes are substantially more complex, especially with the partitioning of metabolism among multiple organelles. The combination of improved modelling techniques that incorporate multiple species and compartments, together with a better multi-omics understanding of individual strains and their responses to pathogens alone or in combination, will thus help us design robust communities for efficient conversion of sunlight into biofuels.

## References

[b402] Beckmann J, Lehr F, Finazzi G, Hankamer B, Posten C, Wobbe L, Kruse O (2009). Improvement of light to biomass conversion by de-regulation of light-harvesting protein translation in *Chlamydomonas reinhardtii*. J Biotechnol.

[b408] Freilich S, Zarecki R, Eilam O, Segal ES, Henry CS, Kupiec M (2011). Competitive and cooperative metabolic interactions in bacterial communities. Nat Commun.

[b404] Gachon CM, Sime-Ngando T, Strittmatter M, Chambouvet A, Kim GH (2010). Algal diseases: spotlight on a black box. Trends Plant Sci.

[b401] Ort DR, Melis A (2011). Optimizing antenna size to maximize photosynthetic efficiency. Plant Physiol.

[b406] Prince EK, Myers TL, Naar J, Kubanek J (2008). Competing phytoplankton undermines allelopathy of a bloom-forming dinoflagellate. Proc Biol Sci.

[b405] Rajamani S, Bauer WD, Robinson JB, Farrow JM, Pesci EC, Teplitski M (2008). The vitamin riboflavin and its derivative lumichrome activate the LasR bacterial quorum-sensing receptor. Mol Plant Microbe Interact.

[b407] Vardi A, Eisenstadt D, Murik O, Berman-Frank I, Zohary T, Levine A, Kaplan A (2007). Synchronization of cell death in a dinoflagellate population is mediated by an excreted thiol protease. Environ Microbiol.

[b403] Yizhak K, Benyamini T, Liebermeister W, Ruppin E, Shlomi T (2010). Integrating quantitative proteomics and metabolomics with a genome-scale metabolic network model. Bioinformatics.

# Article commentary: Know thy microorganism – why metagenomics is not enough!

**Published:** 2012-12-18

**Authors:** Anna Schnürer

**Affiliations:** Department of Microbiology, BioCenter, Swedish University of the Agricultural SciencesBox 7025, 750 07 Uppsala, Sweden

Microorganisms are the dominating species on our planet. Their wide metabolic capacity makes them essential for the cycling of nutrients in the nature and vital for the function of plant growth and animal gut ecosystems. Moreover, microorganisms are used in a multitude of traditional and modern biotechnological applications. In a future sustainable society, with a need for a decreased consumption of fossil carbon sources, the use of microorganism needs to be intensified for making a variety of different organic building blocks, e.g. for pharmaceuticals, as well as for the production of different energy sources. The question then arises, how do we find these new microbial resources?

Most of the knowledge we have today about microorganisms, their physiological capacities and possibilities to use for biotechnological applications has arisen from traditional microbiological methodologies, i.e. the isolation and cultivation of pure strains and species. For many years these methods represented the main tools for generating knowledge about microbial life. Today we have access to powerful tools providing detailed information about the genomic structure and gene expression of isolated microorganisms, giving valuable insights about microbial metabolism and pointing towards new biotechnological possibilities. These new technologies also allow for metagenomic analyses of various complex environments and with comparative sequence analyses we can answer questions related to evolution and metabolic functions, as well as reveal information about previously unknown microbial life. Furthermore, by screening different environments we find patterns and can see connections between environmental conditions and microbial composition and genetic expressions. This is all great and fantastic and we learn a lot! However, what it also obvious from the results is that a majority of the microbial life that is detected relates to unknown microorganisms.

The biogas process is one example of a highly complex microbial environment. This is an amazing microbial process with a great functional capacity. It includes both generalist and specialists, together forming a microbial community that by intricate interactions converts large organic macromolecules into a variety of smaller organic components and finally into methane. The great functional diversity of this process suggests it as a possible source for new organisms to be used for biotechnological applications. I will use this process as a way to exemplify why I think molecular microbial ecologist to a greater extent have to consider also using a traditional route for their research in order to find new and important microbial resources. The biogas process has been used for more than a hundred years for small-scale production of energy for rural households. Today the process is also used in industrial countries for large-scale production of biogas from a variety of different urban and rural waste streams, as well as from dedicated energy crops. In my mind, the breakthrough for the industrial use of this process was a greater understanding of the microbiology behind the process, possible to obtain only after the invention of techniques for cultivation of strict anaerobes (Hungate, 1950). We now have a reasonable understanding of the overall microbial flow in this system and what is required for management to reach sufficient biogas production. Even so, the functionality can still be improved and many questions remain to be answered in order to reach higher stability and efficiency with economic and environmental outcome. Many investigations in this research field, in common with many other fields of microbial ecology, are devoted to different levels of community analysis. We are all fascinated by the new techniques and their possibilities. However, in my opinion there is a great risk that we will get lost in the giant maze of sequences. How can we understand and resolve issues about functional capacity and management and find new organisms for biotechnological applications when a major part of the targeted microbial life is represented by unknown microorganisms (Zakrzewski *et al*., 2012)! Furthermore, critical functions can also be performed by key players present at low abundances and thus not even targeted with the present general community analysis approaches. I might be a bit conservative, but we have also to go back to the methodologies used in the beginning of the microbiology era and put much more resources and efforts into isolation and characterization of new organism.

Without this knowledge we will not come further in linking the population structures to their functional capacities. Nor will we find new organisms with functions of interest, or ways to optimally manage a complex microbial ecosystem such as the biogas process. Furthermore, the scientific community needs to appreciate and value the hard work of microbial isolations and characterization. Today a paper describing the overall composition of a community, using the latest sequencing technology, results in a publication in a high-impact journal, while a paper describing a new organism, with a real potential to have a high impact on science, commonly only appears in low-impact journals. It is thus not surprising that the young microbiologist of today hardly knows how to cultivate an organism! I remain convinced that the way to go for the future work in the field is by using a combination of the techniques. With new isolations we can improve our tools and understanding of the complex community (Westerholm *et al*., 2011) and with metagenomic analysis we might also find new keys for successful isolations of yet unkown microorgaisms (Pope *et al*., 2011). With new isolates we can then study physiology, metabolism, genetics, as wells as microbial interactions and obtain a greater understanding of the microorganisms and their role in the various ecosystems and their potential in a range of applications.

## References

[b501] Hungate RE (1950). The anaerobic mesophilic cellulolytic bacteria. Bacteriol Rev.

[b504] Pope PB, Smith W, Denman SE, Tringe SG, Barry K, Hugenholtz P (2011).

[b503] Westerholm M, Dolfing J, Sherry A, Neil ND, Head IM, Schnürer A (2011). Quantification of syntrophic acetate-oxidizing microbial communities in biogas processes. Environ Microbiol Rep.

[b502] Zakrzewski M, Goesman A, Jaenicke S, Jünemann S, Eikmeyer F, Szczepanowski R (2012). Profiling of the metabolically active community from a production-scale biogas plant by means of high-throughput metatranscriptom sequencing. J Biotechnol.

# Article commentary: Spatial structuring of microbial communities – at the micro- to millimetre scale

**Published:** 2012-12-18

**Authors:** Barth F Smets

**Affiliations:** Department of Environmental Engineering, Technical University of DenmarkDK-2800 Kgs, Lyngby, Denmark

While it is often observed that microbial communities display a surprisingly defined spatial structure, with different community members being located at specific locations relative to one another, this feature is not explicitly targeted when engineering microbial communities for biotechnological applications. Here, I argue that such directed effort at spatially structuring microbial communities at the micro-scale may be within reach, and can provide unique opportunities to assemble microbial communities with specific process objectives, using natural, engineered, or *de novo* synthesized microbes.

Microbes in natural environments typically occur as surface associated communities, often termed biofilms: dense assemblages of cells with different physiologies, which are enmeshed in an exopolymeric substance that provides cohesion, connected to a biotic or abiotic substratum. Because solute transport is typically retarded in a biofilm environment in comparison to a true liquid environment, solute gradients are often encountered. This is especially the case for reactive solutes: substances that are produced or consumed by the microbes. The outcome of these gradients is that microbes with different solute preferences are located at different locations in such biofilm. As a result, in all but the simplest laboratory systems, spatially structured biofilms are the norm. This type of spatial stratification can even take place in simple biofilms, housing only one functional type, where electron donor, electron acceptor, or pH gradients (often a side product of microbial activity), will result in stratification of subpopulations with different affinities or tolerances for said solutes. Stratification is more remarkable and easier discernable when conditions allow for microbial foodwebs, where metabolic end-products of individual guilds generate substrates for other guilds. Here, over distances of less than a few millimetres, an array of different physiological groups, located in defined positions with respect to each other, are necessary to yield a community's metabolism. Typical examples are the spatially structured anaerobic foodwebs where fermentative, acidogenic, and acetogenic metabolisms are connected to respiratory guilds that use CO_2_ or SO_4_^2−^ as terminal electron acceptor. In these examples, it is essential to point out that the community's overall metabolism is contingent on the spatial structuring and that destabilization of the structure results in performance failure. Another example, of long-term interest to me, are structured biofilms or bioaggregates for autotrophic ammonium conversion: here with control of electron acceptor and electron donor delivery (O_2_ vs NH_4_^+^-N) spatially structured communities can be built where within less than a mm, obligate oxygen requiring and oxygen inhibited microbes can coexist to generate an interesting community metabolism (Vlaeminck *et al*., 2010).

Natural examples of such spatially structured or stratified communities are rife, and span scales (mm to km), domains of life, functional types, and environments. In nature, stratification is caused by niche opportunity: microbes position themselves, where things are best for them. But can we co-opt this process to design and build microbial communities we wish? The case of autotrophic N removal in redox-stratified biofims and bioaggregates provides one example: a combination of controls (fluxes of oxygen vs ammonium; potentially supplemented with counter diffusion to set relative position of groups with different redox preferences, and interventions to eliminate unwanted microbes) yields an engineered stratified community, recruited from open communities, consisting of wanted metabolic guilds (Nacher *et al*., 2010). This concept of *sorting* a target community from a diverse mixed community, can be repeated for other systems (e.g. anaerobic foodwebs) where spatial proximity, external control and imposition of gradients can be used to steer competition within a microbial community, weeding out community members, and causing a more structured microbial community.

I contend that spatial arrangements should also be explored to develop microbial communities *de novo*. In the effort to construct synthetic microbes for great biotechnological purposes (generation of novel chemistry, energy, or biology) it is often recognized that one microbe is not sufficient to host the complete metabolism, but that partitioning of the synthetic metabolism and metabolic load over multiple microbes is preferable to optimize throughput. Creative solutions are being devised to stabilize such synthetic microbial communities (in suspension); they often rely on engineering intercellular communication and dependencies based on synthetic circuits involving quorum sensing signals (Brenner *et al*., 2008). Because these signals are diffusible molecules, spatial stratification might be a further option to stabilize these interactions: ensuring the correct proximal physical relationship of the targeted proximal metabolism. This stratification might also be built by taking advantage of typical gradients in biofilms using hosts with distinct intrinsic or engineered preferences for the gradient solute, or by physically constraining their spatial arrangements. Choosing microbes with differential preferences for electron acceptors or engineering pathways whose expression are responsive to different redox conditions, are obvious options. Direct physical constraints can be provided by artificial immobilization (e.g. on a substratum) or separating (e.g. using non-reactive permeable membranes) of community members. Micropatterning substrata with different surface properties could provide vicinal positions of different microbes, chosen to have the correct combination of metabolism and surface chemistry.

Whether the targeted community is based on engineered or naturally existing metabolic or signalling relationships between microbes, 3-D modelling frameworks have recently become available (e.g. Lardon *et al*., 2011) to test the feasibility of community stratification at the relevant scale, its dependence on environmental or operational conditions, and its effect of the biotechnological system performance. Such modelling efforts can streamline and guide the experimental investigations.

In closing, while microbial community *composition* is a major indicator of a community's metabolic potential, the *spatial structure* of that community, imposed or inherently present, dictates it extant activity. There are opportunities to steer the spatial structure of open and synthetic microbial communities to select or stabilize a target community metabolism from its constituent members. Taking advantage of this microspatial engineering can offer new biotechnological opportunities.

## References

[b603] Brenner K, You L, Arnold FH (2008). Engineering microbial consortia: a new frontier in synthetic biology. Trends Biotechnol.

[b604] Lardon L, Merkey B, Martins S, Dötsch A, Kreft JU, Picioreanu C, Smets BF (2011). iDynoMiCS: next generation individual-based modelling of biofilms. Environ Microbiol.

[b602] Nacher CP, Sun S, Ni B-J, Terada A, Smets BF (2010). Sequential aeration of membrane-aerated biofilm reactors for high-rate autotrophic nitrogen removal: experimental demonstration. Environ Sci Technol.

[b601] Vlaeminck E, Terada A, Smets BF, De Clippeleir H, Schaubroeck T, Bolca S (2010). Aggregate size and architecture determine biomass activity for one-stage partial nitritation and anammox. Appl Environ Microbiol.

# Article commentary: Bacteriophages shaping microbial communities for good and for bad

**Published:** 2012-12-18

**Authors:** E J Smid, T Abee

**Affiliations:** Laboratory of Food Microbiology, Wageningen University6700 EV, Wageningen, The Netherlands

Bacteriophages constitute the most abundant biological entities on our planet (Chibani-Chennoufi *et al*., 2004) and are thought to play a major role in maintaining genetic diversity in microbial communities and consequently affect ecosystem functionality (Weinbauer and Rassoulzadegan, 2004; Rodriguez-Valera *et al*., 2009). Through selective predation by virulent phages and the activity of temperate phages, the composition and activity of microbial communities is continuously challenged. In this contribution, we discuss the options and weigh the chances for using bacteriophages in order to improve biotechnological processes driven by microbial cultures and to trace and control undesirable microbial activity in industrial and medical settings.

In the arena of microbial biotechnology, and specifically at those places where microbial diversity has been abandoned by using simple and defined cultures – for the sake of improved process control – bacteriophages are usually regarded as a threat. For example, producers of starter cultures for dairy applications and their customers (the dairy companies) developed over the years various strategies to suppress the problem of uncontrolled bacteriophage outbreaks in their production environment (Moineau, 1999). On the other hand, the application of bacteriophages as therapeutic tools to combat infectious diseases has been a promise for decades but phage-based methodologies still await widespread acceptance. Renewed interest in phage therapy has come with the recognition that knowledge of the population and evolutionary dynamics of bacterial–phage interactions are crucial for the development effective phage therapy (Levin and Bull, 2004; Hunter, 2012). Understanding the complex nature of prey–predator relationships in the microbial world of infectious diseases is thought to deliver strategies to overcome failure of phage therapy due to the emergence of resistant pathogens. Recently, we can also observe an increased interest in the use of cocktails of bacteriophages for the control of foodborne pathogens. This includes the simple use of bacteriophage encoded endolysins as antimicrobial agents (Oliveira *et al*., 2012) as well as the use of intact lytic phages. Two products, based on cocktails of anti-listerial phages for controlling *Listeria monocytogenes* on ready-to-eat food (Bren, 2007) and meat and cheese products (Carlton *et al*., 2005), have been approved by the FDA and were subsequently introduced in the market. Finally, in the field of environmental biotechnology, for instance in waste water treatment systems using activated sludge, the role of bacteriophages in controlling bacterial abundance, activity, and community composition has been underestimated for a long time (Shapiro and Kushmaro, 2011). For example, a metagenome analysis of activated sludge revealed antimicrobial resistance genes in the bacterial and viral metagenome indicating either the transfer of these genes between bacterial hosts sharing a common phage or lysogenic phages conferring antibiotic resistance to infected cells (van Oppen *et al*., 2009; Parsley *et al*., 2010).

A description of the above-mentioned developments follows current trends in the arena of phage research. However, gazing into our crystal ball, we see two important developments, one related to a better understanding of the role of phages in pathogen ecology and virulence, and the other related to the use of phages in biotechnological and medical settings.

Bacteriophages in lysogenic state, or prophages, have recently been shown to modify pathogen phenotypes thereby affecting environmental performance, conceivably steering selection and evolution. For example, Schuch and Fischetti (2009) showed that lysogeny in *Bacillus anthracis* can affect sporulation capacity, exopolysaccharide synthesis, biofilm formation, and colonization of a range of environments including soil and host organisms. Several phenotypic changes were found to require the expression of phage-encoded bacterial sigma factors. *L. monocytogenes* niche adaptation, biofilm formation and persistence has recently been suggested to be associated with *comK*-prophage-containing genotypes, that show enhanced transduction and recombination (Verghese *et al*., 2011). In line with these observations, co-evolution with lytic phages was found to select for the mucoid phenotype of *Pseudomonas fluorescens* (Scanlan and Buckling, 2012). This phenotype is due to overproduction of alginate and is regarded a considerable virulence factor contributing to intractability of infections most notably in cystic fibrosis (CF) (Høiby *et al*., 2010). Identification of underlying mechanisms will further add to our understanding of pathogen behaviour, and could also help to identify transmission routes and implement more efficient intervention strategies. Furthermore, phage-mediated genetic exchange can affect pathogen virulence, and is assumed to occur primarily within species. However, Chen and Novick (2009) provided evidence that toxin-carrying staphylococcal pathogenicity islands, containing superantigen genes and other mobile elements transferred to *L. monocytogenes* at the same high frequencies as they transfer within *Staphylococcus aureus*. Notably, the phages involved did not form plaques on any of the seven serotypes of *L. monocytogenes* tested, thus the true overall host range of a phage may be much wider than anticipated. This suggests that phages participate in a far more expansive network of genetic information exchange among bacteria of different species than originally thought, with obvious implications for the evolution of (human) pathogens.

We foresee application of the obtained insights to attenuate the behaviour of (foodborne) pathogenic microbes in a variety of environmental niches such as biofilms in industrial and medical settings, including the mammalian GI-tract.

Concerning biotechnological applications, bacteriophages potentially can act as functional members in complex microbial starter cultures – for instance for use in industrial and food fermentations – which possess an unprecedented high degree of functional robustness (Smid and Lacroix, 2013). If we consider the ecological role of bacteriophages in complex microbial communities, i.e. preservation of diversity in prokaryotic populations (Rodriguez-Valera *et al*., 2009), we see potential for using selected bacteriophage cocktails in designed multi-train starter cultures. By adding phages to a balanced mix of phage-resistant and phage-sensitive variants, each carrying the genes to deliver the required product functionality, we reconstitute a dynamic microbial ecosystem with active prey–predator interactions between phages and host microbes, ensuring the conservation of all required gene-functions at community level. Such reconstituted, phage containing complex cultures are expected to retain their functionality while coping with environmental fluctuations. This radical new approach to design complex cultures of microbes, requires deepening of basic and quantitative knowledge of the role of bacteriophages in complex microbial populations. Such knowledge is now, more than ever before, accessible by combining high-throughput metagenome sequencing with mathematical modelling of microbial population dynamics and prey–predator relationships. This development lines up with the current interest in the fundamental architectural features of the behaviour of complex systems such as ecological networks (Scheffer *et al*., 2012). We envision a bright future for research on bacteriophages shaping microbial communities for good and bad, and the implementation of derived insights in biotechnology applications in industrial and medical settings.

## References

[b701] Bren L (2007). Bacteria-eating virus approved as food additive. FDA Consum.

[b702] Carlton RM, Noordman WH, Biswas B, de Meester ED, Loessner MJ (2005). Bacteriophage P100 for control of *Listeria monocytogenes* in foods: genome sequence, bioinformatic analyses, oral toxicity study, and application. Regul Toxicol Pharmacol.

[b703] Chen J, Novick RP (2009). Phage-mediated intergeneric transfer of toxin genes. Science.

[b704] Chibani-Chennoufi S, Bruttin A, Dillmann ML, Brussow H (2004). Phage–host interaction: an ecological perspective. J Bacteriol.

[b706] Høiby N, Ciofu O, Bjarnsholt T (2010). *Pseudomonas aeruginosa* biofilms in cystic fibrosis. Future Microbiol.

[b705] Hunter P (2012). The return of the phage: technological advances enable scientists to engineer and use phages to treat a wide range of diseases including cancer. EMBO Rep.

[b707] Levin BR, Bull JJ (2004). Population and evolutionary dynamics of phage therapy. Nat Rev Microbiol.

[b708] Moineau S (1999). Applications of phage resistance in lactic acid bacteria. Antonie Van Leeuwenhoek.

[b709] Oliveira H, Azeredo J, Lavigne R, Kluskens LD (2012). Bacteriophage endolysins as a response to emerging foodborne pathogens. Trends Food Sci Technol.

[b710] Parsley LC, Consuegra EJ, Kakirde KS, Land AM, Harper WF, Liles MR (2010). Identification of diverse antimicrobial resistance determinants carried on bacterial, plasmid, or viral metagenomes from an activated sludge microbial assemblage. Appl Environ Microbiol.

[b711] Rodriguez-Valera F, Martin-Cuadrado AB, Rodriguez-Brito B, Pai L, Thingstad TF, Rohwer F, Mira A (2009). Explaining microbial population genomics through phage predation. Nat Rev Microbiol.

[b712] Scanlan PD, Buckling A (2012). Co-evolution with lytic phage selects for the mucoid phenotype of *Pseudomonas fluorescens* SBW25. ISME J.

[b713] Scheffer M, Carpenter SR, Lenton TM, Bascompte J, Brock W, Dakos V (2012). Anticipating critical transitions. Science.

[b714] Schuch R, Fischetti VA (2009). The secret life of the anthrax agent *Bacillus anthracis*: bacteriophage-mediated ecological adaptations. PLoS ONE.

[b715] Shapiro OR, Kushmaro A (2011). Bacteriophage ecology in environmental biotechnology processes. Curr Opin Biotechnol.

[b716] Smid EJ, Lacroix C (2013). Microbe-microbe interactions in mixed culture food fermentations. Curr Opin Biotechnol.

[b717] Van Oppen MJH, Leong JA, Gates RD (2009). Coral-virus interactions: a double-edged sword?. Symbiosis.

[b718] Verghese B, Lok M, Wen J, Alessandria V, Chen Y, Kathariou S, Knabel S (2011). *comK* prophage junction fragments as markers for *Listeria monocytogenes* genotypes unique to individual meat and poultry processing plants and a model for rapid niche-specific adaptation, biofilm formation, and persistence. Appl Environ Microbiol.

[b719] Weinbauer MG, Rassoulzadegan F (2004). Are viruses driving microbial diversification and diversity?. Environ Microbiol.

# Article commentary: Will test tubes ever undergo division?

**Published:** 2012-12-18

**Authors:** Miguel Vicente

**Affiliations:** Centro Nacional de Biotecnología, Consejo Superior de Investigaciones Científicas (CNB-CSIC)C/ Darwin n° 3, E-28049, Madrid, Spain

Conventional wisdom has produced a description of bacterial division in which a Z-ring, guided by two site selection mechanisms, assembles at midcell and recruits a bunch of other proteins to form a divisome. The divisome, possessing a septal peptidoglycan synthesizing complex and connected to other periplasmic and outer membrane proteins, is thought to produce a septum that ultimately splits the cell in two almost equal halves.

Recent research in the field has largely focused on describing the molecular mechanisms responsible for the functions of the divisome and the interactions between its elements. Publications and discussions at recent meetings on bacterial division stress the need to further investigate the process to obtain a more exact knowledge on these mechanisms. Although it should not be expected that septation will occur in a test tube, after all cell division works on cells, the recent and future attempts to reconstruct the divisome from its parts will likely help to clarify some of these questions (Martos *et al*., 2012). On the other hand, they may also evidence that some parts may still be missing from the currently accepted picture of the division process.

## Bacterial division: past and future

Cell division in present day bacteria is likely to be a composite process originated at different times in the bacterial phylogeny. A primitive partition system to pinch the membrane may coexist with a more modern mechanism to divide bacterial cells possessing a rigid peptidoglycan layer. Evidence of the ancestral mechanism to divide membranes can be observed in mutant cells devoid of peptidoglycan and osmotically stabilized. These so-called L forms apparently divide as a consequence of imbalances in their surface to volume ratio when the amount of membrane is excessive (Mercier *et al*., 2012). In contrast, in intact bacteria the membrane may be constantly pushed by turgor pressure. This may prevent constriction to happen at random unless a peptidoglycan-containing septum is formed at a specific place. A first topic to be investigated in the future is how septation proceeds as an equilibrium in which the synthesis of peptidoglycan stabilizes the opposing forces of turgor and constriction exerted on the membrane.

## Z-ring, division ring and divisome

The term Z-ring, proposed by Joe Lutkenhaus (Bi and Lutkenhaus, 1991), designates a structure present at midcell of dividing cells and visualized by microscopy techniques using FtsZ fusions to fluorescent proteins or antibodies against it. It is postulated to be a ring of variable diameter formed by the accumulation of FtsZ-containing complexes. The Z-ring may provide a scaffold on which the remaining division elements assemble due to their ability to interact directly or indirectly with FtsZ. In addition FtsZ is prevented from forming rings at the cell poles by complex site selection mechanisms, among them the MinCDE proteins (de Boer *et al*., 1989) and nucleoid occlusion (Cho *et al*., 2011).

The division ring is a multiprotein complex whose existence has been proposed based on the observation that several proteins (e.g. FtsZ, FtsA, ZipA, FtsN) are independently visualized as individual rings at midcell of dividing cells (reviewed in Vicente *et al*., 2006).

Nanne Nanninga proposed the name divisome to define the complete macromolecular machinery able to effect division in the living cell (Nanninga, 1998). Besides the proteins that form the division ring, additional components as periplasmic and outer membrane proteins and the cell envelope may also be integral parts of the divisome.

## The structure of the ring

A wealth of fluorescence microscopy images have been obtained from cells overproducing putatively active fusions of division proteins to fluorescent reporter proteins. Although some fusions may not behave exactly as the wild-type protein (Swulius and Jensen, 2012), their images have been interpreted as indicating that the shapes adopted by divisome and site selection proteins are mostly rings or helixes. This has provided a convenient conceptual framework in which proteins that are assumed to act at midcell (e.g. FtsZ) would naturally form rings and those acting along the length of the cell (e.g. MreB) would wind into helixes.

The molecular structure of the Z ring complex has nevertheless not been firmly identified, and no particular assembly of FtsZ polymers has been unequivocally associated with the material evidenced in the microscopic images of the division ring. High-resolution imaging has recently put forward a ring image that is not uniform (Strauss *et al*., 2012), what would fit with the expected plasticity of a structure that adapts to a variable size, and possibly a variable geometry, during the septation process. Future work will likely exploit the increasing power of high-resolution techniques as well as *in vitro* reconstruction to produce a detailed picture of the Z ring that may better approximate to its molecular architecture in the cell.

A subset of the division ring formed by FtsZ, FtsA and ZipA has been called proto-ring because it is assumed to be the initial step of divisome assembly (Vicente and Rico, 2006). Recently some of the properties of the proto-ring components other than FtsZ have been investigated, they are compatible with their assumed role as anchors of the FtsZ protein to the cytoplasmic membrane (Skoog and Daley, 2012). The availability of streptococcal FtsA, a more manageable form of the protein than its *E. coli* counterpart, offers new opportunities for studying its role on division in the test tube and may also help in defining the proto-ring in Gram-positive cocci (Krupka *et al*., 2012).

## Oscillation of a ring inhibitor

Equally fitting into the conventional description of septum site selection is the pole-to-pole oscillatory behaviour of the interacting MinC and MinD (putative inhibitors of the FtsZ ring) directed by a MinE ring. This oscillation is described to create an inhibitory gradient attaining maximum MinCD concentration at both poles (Meinhardt and de Boer, 2012) and may be generated by the displacement of these proteins along the membrane as a wave. In this way FtsZ is, under normal circumstances, blocked from directing the assembly of divisomes at the poles. Results obtained by single molecule imaging of the inhibitor, the MinCD, proteins associated to bilayers indicate that their attachment is transient and, instead of moving directionally, they show a successive exchange between the solution and the membrane (Loose *et al*., 2011). As there is no observable gradient in the solution, why and how this exchange can be directed to propagate as a wave in the living cell remains unknown. As the structure of the MinCD complex is available (J. Löwe, pers. comm.) it will certainly contribute to provide an explanation for the molecular mechanism underlying pole-to-pole oscillation.

## Synthesis of the ring

Some of the presently unknown functions of the divisome may be clarified once we can study them under controlled conditions in divisomes reconstructed in the test tube. This synthetic approach will help, as it happened in the case of DNA replication, to identify elements that may be missing in the picture as well as to better define protein mechanisms and interactions. As an added bonus, it may yield as well new assays based on molecular tools to find novel antimicrobials to combat infection.

Initially, the definition of a minimal divisome may be a taxing task, because the minimal set of genes needed for viable cell division may not coincide with the minimal set of proteins able to reproduce septation in the test tube. However, the advantage offered by the use of *in vitro* systems is that they can be devised to mimic near to natural crowded environments that can be regulated at will. Different combinations of divisome subsets can then be reconstructed and their function can be tested.

Attempts to reconstruct the divisome in simplified systems are now limited to a few instances in which vesicles, either artificially produced or reconstructed from natural membranes, have been used (Osawa *et al*., 2008; Jiménez *et al*., 2011). Developments along these lines will likely come from the use of more sophisticated and controllable containers in which subsets of the site selection complexes, the constriction mechanisms and combinations of both can be assembled and their function tested. The more complex of these containers may be cells in which the chromosome has been removed, while nanodiscs (Hernández-Rocamora *et al*., 2012), in which proteins that attach to the membrane can be handled in solution, may be the simplest ones. Their use will help to obtain a full molecular description of how bacteria, the most simple cells, manage to precisely control the timing and the topology of division.

## References

[b801] Bi E, Lutkenhaus J (1991). FtsZ ring structure associated with division in *Escherichia coli*. Nature.

[b803] de Boer PA, Crossley RE, Rothfield LI (1989). A division inhibitor and a topological specificity factor coded for by the minicell locus determine proper placement of the division septum in *E. coli*. Cell.

[b802] Cho H, McManus HR, Dove SL, Bernhardt TG (2011). Nucleoid occlusion factor SlmA is a DNA-activated FtsZ polymerization antagonist. Proc Natl Acad Sci USA.

[b804] Hernández-Rocamora VM, Reija B, García C, Natale P, Alfonso C, Minton AP (2012). Dynamic interaction of the *Escherichia coli* cell division ZipA and FtsZ proteins evidenced in nanodiscs. J Biol Chem.

[b805] Jiménez M, Martos A, Vicente M, Rivas G (2011). Reconstitution and organization of *Escherichia coli* proto-ring elements (FtsZ and FtsA) inside giant unilamellar vesicles obtained from bacterial inner membranes. J Biol Chem.

[b806] Krupka M, Rivas G, Rico AI, Vicente M (2012). Key role of two terminal domains in the bidirectional polymerization of FtsA protein. J Biol Chem.

[b807] Loose M, Fischer-Friedrich E, Herold C, Kruse K, Schwille P (2011). Min protein patterns emerge from rapid rebinding and membrane interaction of MinE. Nat Struct Mol Biol.

[b808] Martos A, Jiménez M, Rivas G, Schwille P (2012). Towards a bottom-up reconstitution of bacterial cell division. Trends Cell Biol.

[b809] Meinhardt H, de Boer PA (2001). Pattern formation in *Escherichia coli*: a model for the pole-to-pole oscillations of Min proteins and the localization of the division site. Proc Natl Acad Sci USA.

[b810] Mercier R, Domínguez-Cuevas P, Errington J (2012). Crucial role for membrane fluidity in proliferation of primitive cells. Cell Rep.

[b812] Nanninga N (1998). Morphogenesis of *Escherichia coli*. Microbiol Mol Biol Rev.

[b813] Osawa M, Anderson DE, Erickson HP (2008). Reconstitution of contractile FtsZ rings in liposomes. Science.

[b814] Skoog K, Daley DO (2012). The *Escherichia coli* cell division protein ZipA forms homodimers prior to association with FtsZ. Biochemistry.

[b815] Strauss MP, Liew ATF, Turnbull L, Whitchurch CB, Monahan LG, Harry EJ (2012). 3D-SIM super resolution microscopy reveals a bead-like arrangement for FtsZ and the division machinery: implications for triggering cytokinesis. PLoS Biol.

[b816] Swulius MT, Jensen GJ (2012). The helical MreB cytoskeleton in *E. coli* MC1000/pLE7 is an artifact of the N-terminal YFP tag. J Bacteriol.

[b817] Vicente M, Rico AI (2006). The order of the ring: assembly of *Escherichia coli* cell division components. Mol Microbiol.

[b818] Vicente M, Rico AI, Martínez-Arteaga R, Mingorance J (2006). Septum enlightenment: the assembly of the bacterial division proteins. J Bacteriol.

# Article commentary: I C1 C1-Future

**Published:** 2012-12-18

**Authors:** Karsten Zengler

**Affiliations:** Department of Bioengineering, University of California San DiegoSan Diego, USA, and, The Novo Nordisk Foundation Center for Biosustainability, Technical University of DenmarkLyngby, Denmark

You do not need a crystal ball to envision that an economy that is solely based on fossil fuels provides for an economically and environmentally uncertain future. Passing by my local gas station this morning on my way to the office in San Diego provided a glimpse into this kind of future. As I am writing this piece, Southern California is attempting to break every price record at the pump, thus serving as a foreshadowing of things to come. It is a much more difficult task to envision an alternative reality – a reality in which we turn former mistakes to our advantage, one in which we reduce carbon emissions, conserve water, and utilize energy efficiently without interfering with food supplies. To address this challenge, a crystal ball might actually come in handy. Taking a glance into a archetypical crystal ball you see swirling white smoke resembling the effect created by evaporating dry ice, and all of a sudden the answer is clear – the smoke itself is the solution!

After over a century of transforming multi-carbon (petroleum) molecules into C1 compounds (carbon dioxide and monoxide), the time seems right to reverse this process by using C1 building blocks to create a multi-carbon economy – a type of reverse engineering project of yet unprecedented proportions. The first steps in this direction were already taken in the 1930s, when methane, the most reduced C1 compound and the major constituent of natural gas, was used and converted to liquid transportation fuels by the Fischer–Tropsch process. However, tied correlation between petroleum and natural gas prices has made this (gas-to-liquid) process, until very recently, economically unattractive and, historically, only came into play when sanctions and embargos forced countries to convert one fossil fuel into derivatives of another. But if you do not want an economy that is reliant upon fossil fuels and its volatility in natural gas prices, an economy must be built on oxidized C1 compounds, such as CO_2_ and CO. CO_2_, the most oxidized form of carbon, obviously requires energy for its transformation into desirable multi-carbon compounds. However, these desirable compounds do not always have to be fuel molecules … Generating alternative fuels is a challenge in itself due to the immense appetite we have generated over the century for cheap transportation fuels. Plants and phototrophic microbes use light energy to reduce carbon dioxide but do so with low efficiency inherent to the photosynthetic systems. Reduction of CO_2_ can also be accomplished using chemical energy (e.g. in form of hydrogen) or electrical energy (Conrado *et al*., 2013). Yet energetically more favourable is the conversion of CO, as it does not require additional energy for its conversion into multi-carbon compounds. High-temperature liquefaction of not only natural gas but also of biomass, and even waste streams and trash, leads to formation of primarily CO and H_2_ (synthesis or syngas). CO can also be generated electrochemically from CO_2_ using catalysts that have high turnover numbers at work at low overpotentials, providing a possible alternative to liquefaction in the future (Rosen *et al*., 2011; Costentin *et al*., 2012). Chemoautotrophic microorganisms are capable of utilizing CO_2_ and H_2_ and some acetogens can even produce simple carbon compounds such as ethanol and acetate from CO alone. Although there have been attempts to use acetogens industrially for the conversion of syngas, until recently, most efforts did not pass the pilot plant stage, in part due to unfavourable mass transfer of CO in traditional bioreactors and the low value of acetate – the major product of acetogenesis. However, this is changing rapidly with the advent of novel bioreactor designs and, even more importantly, with the ability to genetically engineer these microorganisms, as it has been done successfully for the acetogen *Clostridium ljungdahlii* (Köpke *et al*., 2010). Suddenly higher value products become feasible to produce. Developing design strategies for engineering of microorganisms in the context of the whole cell is the hallmark of genome-scale modelling (Lee *et al*., 2012) and detailed reconstructions for key organisms are currently underway, destined to aid rapid strain design for a C1 economy.

Ultimately, after gaining sufficient knowledge, for example, about acetogens, we might be able to transfer critical energy conservation pathways (e.g. the Rnf complex) and the necessary carbon fixation machinery of the Wood–Ljungdahl pathway into other chassis organisms like *E. coli*. Until then, the genetically tractable autotroph will become the industrial workhorse of choice for building a new biosustainable future … one carbon molecule at a time.

## References

[b901] Conrado R, Haynes C, Haendler B, Toone E, Lee JW (2013). Electrofuels: a new paradigm for renewable fuels. Advanced Biofuels and Bioproducts.

[b903] Costentin C, Drouet S, Robert M, Saveant JM (2012). A local proton source enhances CO_2_ electroreduction to CO by a molecular Fe catalyst. Science.

[b904] Köpke M, Held C, Hujer S, Liesegang H, Wiezer A, Wollherr A (2010). *Clostridium ljungdahlii* represents a microbial production platform based on syngas. Proc Natl Acad Sci USA.

[b905] Lee JW, Na D, Park JM, Lee J, Choi S, Lee SY (2012). Systems metabolic engineering of microorganisms for natural and non-natural chemicals. Nat Chem Biol.

[b902] Rosen BA, Salehi-Khojin A, Thorson MR, Zhu W, Whipple DT, Kenis PJ, Masel RI (2011). Ionic liquid-mediated selective conversion of CO_2_ to CO at low overpotentials. Science.
